# In search of the best method to detect carriage of carbapenem-resistant *Pseudomonas aeruginosa* in humans: a systematic review

**DOI:** 10.1186/s12941-024-00707-1

**Published:** 2024-06-10

**Authors:** Selvi N. Shahab, Anneloes van Veen, Andrea C. Büchler, Yulia R. Saharman, Anis Karuniawati, Margreet C. Vos, Anne F. Voor in ’t holt, Juliëtte A. Severin

**Affiliations:** 1 Department of Medical Microbiology and Infectious Diseases, Erasmus MC University Medical Center, PO Box 2040, 3000 CA Rotterdam, The Netherlands; 2 Department of Clinical Microbiology, Faculty of Medicine, Universitas Indonesia/Dr. Cipto Mangunkusumo General Hospital, Jakarta, Indonesia

**Keywords:** *Pseudomonas aeruginosa*, Carbapenem, Bacterial drug resistance, Culture media, Contact screening

## Abstract

**Background:**

Detection of carbapenem-resistant *Pseudomonas aeruginosa* (CR-PA) in humans is important to prevent transmission. However, the most optimal culture method to detect CR-PA is unknown. This systematic review aims to determine which culture method is most sensitive and which culture methods are used to detect CR-PA in humans. Second, to establish the most feasible culture method taking into account the turnaround time (TAT), and third, to provide an overview of the sampling sites used to detect carriage.

**Methods:**

We systematically searched the electronic databases Embase, Medline Ovid, Cochrane, Scopus, CINAHL, and Web of Science until January 27, 2023. All diagnostic accuracy studies comparing two or more culture methods to detect CR-PA and recent outbreak or surveillance reports on CR-PA carriage or infection in humans, which describe culture methods and their results, were eligible for inclusion. We used QUADAS-2 guideline for diagnostic accuracy studies and the STROBE or ORION guideline for outbreak-surveillance studies to assess the risk of bias.

**Results:**

Six diagnostic accuracy studies were included. An enrichment broth was found to increase the detection of CR-PA. Using an enrichment broth extended the TAT by 18–24 h, yet selective media could reduce the TAT by 24 h compared to routine media. In total, 124 outbreak-surveillance studies were included, of which 17 studies with surveillance samples and 116 studies with clinical samples. In outbreak-surveillance studies with surveillance samples, perianal, rectal swabs or stools were the most common sampling site/specimen (13/17, 76%). A large variety was observed in whether and which kind of enrichment broth and selective media were used.

**Conclusions:**

We found a benefit of using an enrichment step prior to inoculation of the material onto selective media for the detection of CR-PA. More research is needed to determine the most sensitive sampling site and culture method.

*Trail registration*: This study was registered in the PROSPERO International prospective register of systematic reviews (registration number: CRD42020207390, http://www.crd.york.ac.uk/PROSPERO/display_record.asp?ID=CRD42020207390).

**Supplementary Information:**

The online version contains supplementary material available at 10.1186/s12941-024-00707-1.

## Background

*Pseudomonas aeruginosa* is an important healthcare-associated pathogen capable of causing severe infections in hospitalized patients [1, 2]. These infections are often difficult to treat because of the bacteria’s intrinsic and acquired resistance mechanisms [2, 3]. Carbapenem antibiotics are considered to be important agents for the treatment of infections with *P. aeruginosa* [4]. However, the worldwide emergence of carbapenem-resistant *P. aeruginosa* (CR-PA) limits therapeutic options and is associated with high morbidity and mortality [5–7]. Therefore, the World Health Organization (WHO) has marked CR-PA as one of the critical priority pathogens [8].

Hospital outbreaks with CR-PA have been reported globally [9–12]. Even when several infection prevention and control measures are implemented, outbreaks can become large and long-lasting [13, 14]. Water-related reservoirs, such as sinks and showers, play an important role in outbreaks with CR-PA [13, 15, 16]. CR-PA from patients may contaminate and colonize these wet environmental niches and from these reservoirs, CR-PA can spread to other patients. Patient-to-patient transmission can also occur and accounts for almost 14% of transmission events [17]. Additionally, approximately one-third of CR-PA carriers may also develop a clinical infection [18]. This underpins the urgent need for a culture method with high sensitivity and short turnaround time (TAT) to detect CR-PA in humans.

Therefore, this systematic review aimed to determine which culture method is most sensitive and which culture methods are used to detect CR-PA in humans. To that end, two types of studies were of interest: diagnostic accuracy studies comparing different culture methods to detect CR-PA and recent outbreak or surveillance reports describing culture methods to detect CR-PA. For diagnostic accuracy studies, secondary aims were to establish which culture method is most feasible (i.e., storage requirements and product availability) to be implemented in the clinical setting and to determine the TAT for different culture methods. For outbreak-surveillance studies, the secondary aim was to provide an overview of the sampling sites used for screening patients, healthy humans, and/or healthcare workers (HCW).

## Methods

This systematic review was conducted in accordance with the Preferred Reporting Items for Systematic Reviews and Meta-Analyses (PRISMA) statement [19]. The PRISMA 2020 Checklist is available in Supplementary Table S1. This study was registered in the PROSPERO International prospective register of systematic reviews (registration number: CRD42020207390, http://www.crd.york.ac.uk/PROSPERO/display_record.asp?ID=CRD42020207390).

### Search strategy

We systematically searched the electronic databases Embase, Medline Ovid, Cochrane, Scopus, Cinahl, and Web of Science (until January 27, 2023) for studies of any design and distinguished between 1. diagnostic accuracy studies which compared two or more culture methods to detect CR-PA and 2. Outbreak-surveillance studies in which the sampling sites and culture methods used to detect CR-PA were described. Outbreak-surveillance studies were included if published between September 7, 2019, and January 27, 2023, in order to establish an overview of the most recently used methods. The search was not limited by language or country of publication (Supplementary File S1).

For both types of studies, we excluded conference abstracts, letters to the editor, and brief communications. Additionally, we excluded studies reporting on culture methods for blood samples only, since these methods (e.g., initial incubation in the BACTEC) are inherently different from the methods used for other types of samples. For outbreak-surveillance studies, we excluded studies in which only a description of the culture methods used for species re-identification was provided, and no additional clarification on the initial culture methods could be retrieved from corresponding authors. Moreover, reference lists of reviews of interest were screened to identify additional studies initially missed by our search strategy.

Titles and abstracts of all retrieved citations were screened independently by SNS and AvV/ACB. After this screening, SNS and AvV/ACB performed a second screening based on the full text. Disagreements were resolved by discussion, involving a third reviewer (AV), if required.

### Definitions

The current body of literature does not always clearly differentiate between CR-PA carriage and infection. Therefore, we initially included all relevant outbreak-surveillance studies describing CR-PA detection and, subsequently, distinguished between carriage and infection. The following definitions were used, which may differ from the terminology used in included studies; Carriage is defined as the presence of CR-PA (often) in a non-sterile body site and the absence of clinical symptoms. Surveillance samples are used to detect CR-PA carriage. Infection is defined as the presence of CR-PA in a sterile body site (most often) with clinical symptoms for which treatment is needed. Clinical samples are used to detect CR-PA infection.

The feasibility of a culture method was determined by the storage requirements (e.g., storage at low temperatures necessitates the use of a refrigerator) and availability of the products used. It was checked whether products were claimed as “available worldwide” from the manufacturers’ websites and subsequently, it was cross-checked if they could be ordered in the Netherlands and Indonesia as indication of worldwide availability. When the product could not be ordered in at least one of these two countries for any reason, it was concluded that it was not available worldwide as claimed.

### Data extraction and analysis

At least two reviewers (SNS, AvV/ACB) independently extracted data from individual records using predefined templates. We collected data on study design, year of publication, country of publication, and culture methods for all included studies. Additionally, for diagnostic accuracy studies and outbreak-surveillance studies with surveillance samples, we collected data on healthcare setting, study population, number of samples (overall and positive for CR-PA), and body sites tested. Measures of test accuracy were also collected from diagnostic accuracy studies. Corresponding authors of included studies were requested to approve the extracted data and to provide missing data by email.

In case a diagnostic accuracy study did not provide measures of test accuracy for CR-PA specifically, measures were calculated where possible. To determine the TAT (i.e., time between inoculation and confirmation of carbapenem resistance in *P. aeruginosa*), the longest possible duration mentioned was used. All types of antimicrobial susceptibility tests (AST) were scored as having a duration of one day, unless more specific information was provided. PCR for species confirmation was considered to be performed on the same day as AST, unless explicitly stated otherwise.

Data were entered into IBM SPSS version 28 (IBM Corp., Armonk, New York, USA). Missing data were reported as such, whereas incomplete data were used in all analyses. For descriptive purposes, frequencies and percentages were calculated where applicable. Outbreak-surveillance studies using surveillance cultures were analyzed separately from studies using clinical cultures.

### Study quality

SNS, AvV and ACB independently performed the quality assessments of included diagnostic accuracy and outbreak-surveillance studies with surveillance samples. The methodological quality of outbreak-surveillance studies with clinical samples was not assessed since we only extracted microbiological methods from these studies. The methodological quality was assessed using the Quality Assessment of Diagnostic Accuracy Studies (QUADAS-2) guideline for included diagnostic accuracy studies [20] and the Strengthening Reporting of Observational Studies in Epidemiology (STROBE) guideline [21] or the guidelines for transparent reporting of Outbreak Reports and Intervention studies Of Nosocomial infection (ORION) [22] for included outbreak-surveillance studies with surveillance samples. Studies were classified based on threshold values, which were determined by dividing the maximum score possible for each assessment tool in thirds: high; 13–18 points, medium; 7–12 points, and low; 0–6 points for QUADAS-2, high; 23–33 points, medium; 12–22 points, and low; 0–11 points for STROBE, and high; 35–52 points, medium; 18–34 points, and low; 0–17 points for ORION (Table S2).

## Results

**Fig. 1:**
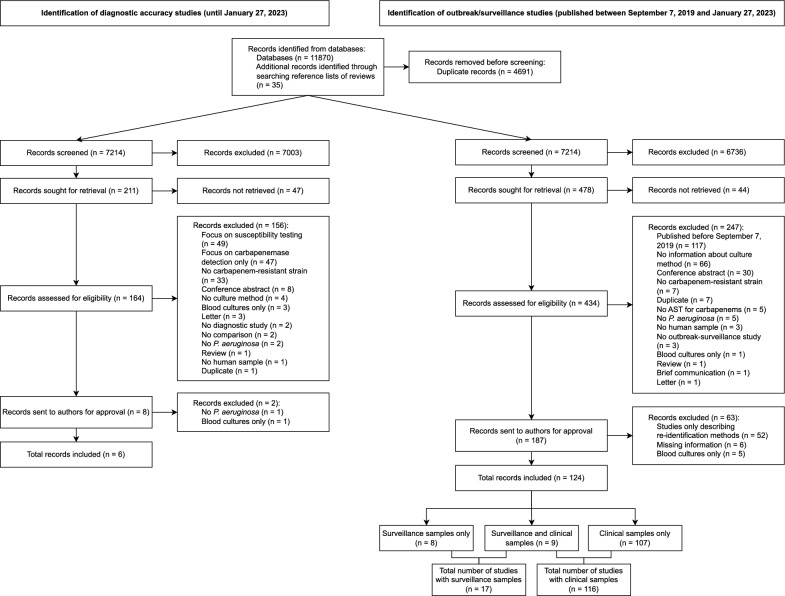
Flowchart for the identification of studies. This figure presents the flowchart of the overall search, which has led to the identification of diagnostic accuracy studies and outbreak-surveillance studies

The search identified 11,870 records from six databases, and 35 additional records were identified from the reference lists of reviews (Fig. 1). Full text screening resulted in the inclusion of 8 diagnostic accuracy studies and 187 outbreak-surveillance studies, of which extracted data was sent to corresponding authors for approval (Fig. 1). The corresponding authors of 56 out of 195 studies (28.7%) approved the extracted data and/or provided missing data. Two diagnostic accuracy studies and sixty-three outbreak-surveillance studies were excluded after (attempts to have) contact with the corresponding author to provide essential missing information. In total, 6 diagnostic accuracy studies and 124 outbreak-surveillance studies, of which 17 studies reported on surveillance samples and 116 studies on clinical samples, were included in this systematic review (Fig. 1).

### Diagnostic accuracy studies

#### Study characteristics

All six included diagnostic accuracy studies used clinical samples for their experiments. Demographic information on these samples was often not reported (Table 1).

**Table 1:** Study characteristics of diagnostic accuracy studies and recent outbreak-surveillance studies with surveillance samples

Study characteristics	Diagnostic accuracy studies (n = 6)	Outbreak-surveillance studies with surveillance samples (n = 17)
	N (%)	N (%)
Study population		
Patients	6 (100)	14 (82)
Healthy persons	0 (0)	0 (0)
HCW	0 (0)	1 (6)
Patients and healthy persons	0 (0)	1 (6)
Patients and HCW	0 (0)	1 (6)
Adults and/or children included		
Adults	1 (17)	10 (59)
Children	0 (0)	0 (0)
Both	3 (50)	3 (18)
Not mentioned	2 (33)	4 (24)
CF patients included		
Yes	3 (50)	0 (0)
No	3 (50)	16 (94)
Not applicable^a^	0 (0)	1 (6)
In-/outpatients included		
Inpatients	0 (0)	14 (82)
Outpatients	1 (17)	1 (6)
Not mentioned	5 (83)	1 (6)
Not applicable^a^	0 (0)	1 (6)
ICU setting		
Yes	0 (0)	11 (65)
No	0 (0)	5 (29)
Not mentioned	6 (100)	1 (6)
Outbreak setting		
Yes	0 (0)	4 (24)
No	5 (83)	13 (77)
Not mentioned	1 (17)	0 (0)
Surveillance and/or clinical samples		
Surveillance samples	0 (0)	8 (47)
Clinical samples	6 (100)	N/A
Surveillance and clinical samples	0 (0)	9 (53)

*CF* cystic fibrosis, *HCW* healthcare workers, *ICU* intensive care unit, *N/A* not applicable

^a^Not applicable for studies including surveillance samples of HCWs only

#### Culture methods

Culture methods were diverse (Table 2). Three studies compared different (chromogenic) selective agars, of which additional information (e.g., storage requirements) is listed in Table S3 [23–25].

**Table 2:** Culture methods described in diagnostic accuracy studies (n = 6)

Reference	Location and year of publication	Methods evaluated	Specimen origin	Use of an enrichment broth	Identification method	Susceptibility test	Use of selective media	Duration
Da Cunha et al. [28]	Brazil, 2020	Reference method: TAT of a final microbiological report (i.e., bacterial identification plus antimicrobial susceptibility testing and detection of carbapenemases) Test method: TAT of bacterial identification and a blue-carba rapid test for carbapenemase detection	Respiratory secretions, urine, blood, and other secretions	Reference method: No Test method: No	Reference method & test method: Identification based on production of characteristic pigments and biochemical tests, such as oxidase production, nitrate reduction and growth in Cetrimide agar	Reference method: Disk diffusion (CLSI) Test method: Blue-carba rapid test	Reference method & test method: Yes, Cetrimide agar	Reference method: Non-carbapenemase producers 3.06 ± 0.71 days, carbapenemase producers 3.19 ± 0.95 days Test method: Non-carbapenemase producers 1.61 ± 0.60 days, carbapenemase producers 1.66 ± 0.89 days
Fournier et al. [27]	Switzerland, 2020	Reference method: direct plating of spiked stools in different dilutions on a selective medium (CHROMagar-Pseudomonas supplemented with 2 µg/mL of meropenem) Test methods: the use of two different enrichment steps (18 h growth in 5 mL TSB or 5 mL TSB supplemented with 1 µg/mL of meropenem) prior to plating spiked stools in different dilutions on a selective medium	Not mentioned	Reference method: No Test methods: Yes, 5 mL TSB (Sigma‒Aldrich, Buchs, Switzerland) or 5 mL TSB (Sigma‒Aldrich, Buchs, Switzerland) supplemented with 1 µg/mL of meropenem	Reference method & test method: Not mentioned (well-characterized collection of strains)	Reference method & test method: Disk diffusion method, MIC values were determined by E-test (bioMérieux) following EUCAST/CLSI joint guidelines	Reference method & test method: Yes, modified CHROMagar-*Pseudomonas* supplemented with 2 µg/mL of meropenem	Reference method: 2 days Test method: 3 days
McMullen et al. [24]	USA, 2017	Reference: well-characterized strain collection. Test method: evaluation of HardyCHROM CRE agar and chromID CARBA agar for the recovery of meropenem-resistant Gram-negative bacteria	Urine, blood, respiratory specimens, body fluids, tissues, and wounds (from frozen stock)	Reference method: Not mentioned Test method: No	Reference method & test method: Not mentioned (well-characterized collection of strains)	Reference method & test method: Disk diffusion method for a subset of strains, for the other strains not mentioned	Reference method: Not mentioned Test methods: Yes, HardyCHROM CRE (Hardy Diagnostics) and chromID CARBA Agar (bioMérieux)	Reference method: Not mentioned Test method: 1–2 days incubation
Laine et al. [23]*	UK, 2009	Reference method: standard identification methods with Columbia blood agar and Pseudomonas CN selective agar Test method: a novel chromogenic medium, PS-ID, for isolation and simultaneous identification of *P. aeruginosa*	Sputum (from CF patients)	Reference method: No Test method: No	Reference method: Inoculation of standard and selective agar plates, oxidase negative isolates were confirmed by API 20 NE, oxidase positives not confirmed as *P. aeruginosa* were subjected to PCR, and isolates not confirmed as *P. aeruginosa* were identified by API 20 NE Test methods: Inoculation of sputum on different plates and observing growth and colonial appearance	Reference method & test methods: Disk diffusion method (on Isosensitest agar) following recommendations from British Society for Antimicrobial Chemotherapy	Reference method: Yes, Pseudomonas CN selective agar and Phenantroline/C-390 agar Test method: Pseudomonas chromogenic medium (PS-ID, bioMérieux, France)	Reference & test method: 6 days
Perry et al. [25]*	UK, 2008	Reference method: routine culture media Test method: selective culture medium (Iso-Sensitest agar supplemented with 4 mg/L of meropenem)	Sputum (from CF patients)	Reference method: No Test method: No	Reference method: inoculation on routine media, including Columbia blood agar supplemented with 5% horse blood, chocolate agar supplemented with 70 mg/L bacitracin, cystine lactose electrolyte deficient agar, *Pseudomonas* CN selective agar, and Iso-Sensitest agar Test method: inoculation on Iso-Sensitest agar supplemented with 4 mg/L meropenem Reference & test methods: All Gram-negative bacteria detected in routine media or selective medium were inoculated on PC agar, Cetrimide agar (Oxoid) and blood agar Isolates growing on all three media were regarded as *P. aeruginosa*. Other strains were confirmed by API 20 NE strips	Reference method & test method: Standard agar dilution method	Reference method: Yes, *Pseudomonas* CN selective agar Test method: Yes, Iso-Sensitest agar supplemented with 4 mg/L of meropenem	Reference & test method: max. 8 days
Zebouh et al [26]*	France, 2008	Reference method: standard procedures of identification followed by antimicrobial susceptibility testing Test method: direct sputum antimicrobial susceptibility testing (DSST, incl. identification) by applying an E-test directly on plates inoculated with sputum (i.e., without a standardized inoculum of a single bacterial species)	Sputum (from CF patients)	Reference method: No Test method: No	Reference method: Inoculation on Cetrimide agar, followed by identification with standard microbiological tests including API 20 NE strips (bioMérieux, Marcy l'Etoile, France) and partial 16S rRNA if necessary Test method: inoculation on blood-supplemented MHA plates in combination with an E-test strip	Reference method: Disk diffusion method (on MHA, bioMérieux, Marcy L’Etoile, France) following CLSI guidelines Test method: E-test	Reference method: Yes, Cetrimide agar Test method: No	Reference method: 3–4 days Test method: max. 2 days

*CF* Cystic Fibrosis, *CLSI* Clinical & Laboratory Standards Institute, *EUCAST* European Committee on Antimicrobial Susceptibility Testing, *MHA* Mueller Hinton agar, *MIC* minimal inhibitory concentration, *PCR* polymerase chain reaction, *TAT* turnaround time, *TSB* tryptic soy broth, *WGS* whole-genome sequencing

^*^Studies reporting on the use of sputum samples from Cystic Fibrosis patients

#### Measures of accuracy

Two different agar plates, the chromID CARBA and HardyCHROM CRE agars, both had 100% sensitivity in a study using 2 drops of 0.5 McFarland standard suspensions of a set of well-defined isolates (Table 3). Zebouh et al., compared antimicrobial susceptibilities as measured by direct sputum antimicrobial susceptibility testing (DSST) by applying an E-test directly on plates inoculated with sputum to standard procedures (identification followed by AST) and found 96.3% agreement, 2.2% very major discordance, 0.8% major discordance, and 0.7% minor discordance for imipenem resistance between both methods using the 2006 CLSI criteria [26]. Furthermore, Fournier et al. found that the use of an enrichment step, irrespective of whether a non-selective or selective enrichment broth was used, prior to inoculation of a selective medium significantly increased the detection of CR-PA compared to direct plating of spiked stools on a selective medium [27].

**Table 3:** Measures of accuracy from diagnostic accuracy studies

Reference	Methods evaluated	Total number of samples/number of positives reference method (% positives)	Total number of samples/number of positives test method(s) (% positives)	Agreement between methods	Sensitivity	Specificity	PPV	NPV
McMullen et al. [24]	Evaluation of two chromogenic agars (HardyCHROM CRE and chromID CARBA Agar) for the recovery of meropenem-resistant Gram-negative bacteria using a set of well-characterized strains	189/19 (10.1) CR-PA, of which 4 CR-PA possess a carbapenemase (*bla*_VIM_) gene and 15 CR-PA do not possess a carbapenemase gene	*HardyCHROM CRE agar: 19/19 strains confirmed chromID CARBA agar: 15/15 confirmed*^*a*^	*HardyCHROM CRE agar: 100% chromID CARBA agar: 100%*^*a*^	*HardyCHROM CRE agar: 100% chromID CARBA agar: 100%*^*a*^	N/A	N/A	N/A
Laine et al. [23]*	Comparison of standard identification methods with Columbia blood agar, Pseudomonas CN selective agar, and a novel chromogenic medium, PS-ID, for isolation and simultaneous identification of *P. aeruginosa*	100/62 (62) *P. aeruginosa*^b^, of which 36 meropenem-resistant *P. aeruginosa*	24 h BA: 100/46 (46.0) *P. aeruginosa* CN: 100/52 (52.0) *P. aeruginosa* PS-ID: 100/36 (36.0) *P. aeruginosa*	24 h BA: not available CN: not available PS-ID: not available	24 hours^c^ BA: 74.1% CN: 83.9% PS-ID: 58.1%	24 h BA: not available CN: not available PS-ID: not available	24 hours^e^ BA: N/A^f^ CN: 89.7% PS-ID: 100%	24 hours^e^ BA: N/A^f^ CN: 76.2% PS-ID: 59.4%
			48 h BA: 100/55 (55.0) *P. aeruginosa* CN: 100/59 (59.0) *P. aeruginosa* PS-ID: 100/58 (58.0) *P. aeruginosa*	48 h BA: not available CN: not available PS-ID: not available	48 hours^c^ BA: 88.7% CN: 95.2% PS-ID: 93.5%	48 h BA: not available CN: not available PS-ID: not available	48 hours^e^ BA: N/A^f^ CN: 88.5% PS-ID: 98.3%	48 hours^e^ BA: N/A^f^ CN: 90.9% PS-ID: 90.2%
			72 h BA: 100/56 (56.0) *P. aeruginosa* CN: 100/59 (59.0) *P. aeruginosa* PS-ID: 100/59 (59.0) *P. aeruginosa*	72 h BA: not available CN: not available PS-ID: not available	72 hours^c^ BA: 90.3% CN: 95.2% PS-ID: 95.2%	72 h BA: not available CN: not available PS-ID: not available	72 hours^e^ BA: N/A^f^ CN: 88.5% PS-ID: 98.3%	72 hours^e^ BA: N/A^f^ CN: 90.9% PS-ID: 92.5%
					Meropenem-resistant *P. aeruginosa*^d^ BA: 83.3% CN: 77.8% PS-ID: 83.3%			
Perry et al. [25]*	Comparison of routine culture media^g^ to a selective culture medium (Iso-sensitest agar supplemented with 4 mg/L of meropenem)	Routine media: 45/42 (93.3) samples positive for *P. aeruginosa* and 30/21 (70) samples positive for CR-PA	Selective media: 45/43 (95.6) samples positive for *P. aeruginosa* and 30/29 (96.7%) samples positive for CR-PA	not available	not available	not available	93% for CR-PA detection^h^	93% for CR-PA detection^i^

Text in italics indicates that numbers were not present in the original article and were, therefore, calculated

*BA* Columbia blood agar, *CN Pseudomonas* CN selective agar, *CPPA* carbapenemase-producing *Pseudomonas aeruginosa*, *CR-PA* carbapenem-resistant *Pseudomonas aeruginosa, N/A* not applicable, *PS-ID Pseudomonas* chromogenic medium, *PPV* positive predictive value, *NPV* negative predictive value

^a^Assuming one non-carbapenemase-producing carbapenem-resistant *P. aeruginosa* grew poorly on chromID CARBA agar

^b^A combination of growth on phenanthroline/C-390 agar, cetrimide agar and growth at 42 °C or species-specific PCR was confirmed for *P. aeruginosa*

^c^Based on the number of samples positive for *P. aeruginosa*

^d^Based on available data, only the sensitivity was calculated

^e^Based on the number of colony variants confirmed as *P. aeruginosa*

^f^Not applicable according to the authors

^g^Routine culture media included Columbia blood agar supplemented with 5% horse blood, chocolate agar supplemented with 70 mg/L bacitracin, cystine lactose electrolyte deficient agar, *Pseudomonas* CN selective agar and Iso-sensitest agar

^h^Indicates the PPV of growth on selective agars to predict antimicrobial resistance as confirmed by MIC testing [25]

^i^Indicates the NPV of the absence of *P. aeruginosa* on selective media to predict susceptibility as confirmed by MIC testing[25]

^*^Studies reporting on the use of sputum samples from Cystic Fibrosis patients

#### TAT

The heterogeneity between the different culture methods led to a broad range of observed TAT (Table 2). Using an enrichment broth was found to significantly increase the detection of CR-PA, but it extended the TAT by 18–24 h [27]. On the contrary, the use of selective media reduced the TAT for the detection of antimicrobial resistance by 24 h compared to routine culture media [25].

#### Study quality

The methodological quality of six diagnostic accuracy studies was assessed by the QUADAS-2 guideline. Five studies were of high quality with scores ranging from 13 to 17 points [24–28], while one study was of medium quality (11 points) [23].

### Outbreak-surveillance studies with surveillance samples

#### Study characteristics

In total, surveillance samples were taken in 17 out of 124 (13.7%) outbreak-surveillance studies. These studies were performed in Europe (n = 9, 53%), Asia (n = 3, 18%), Africa (n = 3, 18%), and South America (n = 2, 12%). Samples were derived from adults in 10 studies (59%) (Table 1). Furthermore, samples were taken from patients in 14 studies (82%), whereas one study (6%) took surveillance samples from patients and healthy persons, one study (6%) from patients and HCW, and one study (6%) from HCW only. Among patients, samples from inpatients were most frequently included (82%). Lastly, four studies (24%) reported on an outbreak setting [16, 29–31].

#### Culture methods of outbreak-surveillance studies with surveillance samples

The majority of studies (n = 13, 76%) used perianal swabs, rectal swabs or stool as body site/specimen for surveillance (Table 4). The most common type of swab reported was the cotton-tipped swab (n = 5, 29%). Six studies (35%) described the use of an enrichment step, with a different broth used in each of those studies. Selective media were used in nine studies (53%), with the *Pseudomonas aeruginosa* Cetrimide agar (n = 3) most often reported. Species identification was (partly) done by automated systems in thirteen studies (76%), and included the use of VITEK (bioMérieux) and matrix-assisted laser desorption/ionization time-of-flight mass spectrometry (MALDI-TOF MS). A direct PCR on surveillance samples for species identification was used in only one study (6%) (personal communication with the corresponding author) [29], whereas PCR for confirmation of *P. aeruginosa* (e.g., after culturing and/or using automated systems for species identification) was used in three studies (18%). Of these, two studies targeted the *oprL* gene and one study targeted the *16S rDNA* gene. One study performed a direct PCR on the broth, in which surveillance samples were initially enriched, to detect *bla*_*VIM*_, after which positive samples were further processed by subculturing the broth on non-selective agar plates [17].

**Table 4:** Methods used in 17 outbreak-surveillance studies with surveillance samples published between 07-09-2019 and 27-01-2023

Reference	Country	Study period	Study population	Sampling sites used for surveillance samples	Use of enrichment broth	Use of selective media	Prevalence (n of persons with CRPA/n of persons sampled)
Adelantado et al. [30]	Spain	January–December 2019	Patients	Perianal swabs	No	Yes, CHROMID® CARBA SMART, CHROMID® ESBL	Not available
Catho et al. [16]	Switzerland	March 2018–September 2020	Patients	Perianal swabs	No	Yes, ChromID OXA-48	Not available
DeGeyter et al. [43]	Belgium	January–December 2019 and October–December 2020	Patients	Rectal swabs	Yes, Fastidious Organisms broth	No	0%^a^
Franco et al. [32]	Paraguay	November 2009–December 2015	Patients	Rectal swabs	No	No	Not available
Freire et al. [29]	Brazil	February 2019–February 2020	Patients	Rectal swabs	Yes, thioglycolate broth	No	5.3%
Hu et al. [44]	China	January 2014–December 2019	Patients	Fecal samples	No	No	1.7%
Karampatakis et al. [45]	Greece	August 2012–November 2016 and December 2016–December 2017	Patients	Rectal swabs	No	Yes, MacConkey agar supplemented with 1 µg/mL meropenem	2.8%
Maclean et al. [46]	South Africa	June 2018–June 2019	Patients	Ear swabs	Yes, nutrient broth	Yes, *Pseudomonas aeruginosa* Cetrimide agar	7.1%
Mahmoud et al. [47]	Egypt	December 2017–March 2020	HCW	Hands	No	No	Not available
Martak et al. [48]	France & Germany	November 2017–April 2019	Patients	Fecal samples	No	Yes, Cetrimide agar plates (Bio-Rad, Marnes-la-Coquette, France)	3.0%
Ohadian Moghadam et al. [49]	Iran	January 2018–January 2020	Patients	Urine	No	No	24.2%
Odoi et al. [50]	Ghana	September 2015–July 2016	Patients and healthy humans	Stool and urine (patients), and hand swabs (farmers)	Yes, soybean-casein-digest broth	Yes, Cetrimide agar (Thermo Fisher) and *Pseudomonas* isolation agar (Alpha Biosciences)	3.0%
Pham et al. [17]	The Netherlands	January 1, 2010–May 18, 2018	Patients	Throat swabs and rectal swabs	Yes, for VIM-PA screening: TSB with 2 mg/L ceftazidime and 50 mg/L vancomycin	Yes, for SDD screening: ESBL CHROMagar plate (BD diagnostics, Breda, the Netherlands) or CHROMID ESBL (bioMérieux, Marcy l'Etoile, France)	0.8%
Rice et al. [31]	England	September 2016–November 2020	Patients	Throat swabs, rectal swabs, and wounds (including line sites)	No	Yes, msuperCARBA (Oxoid)	Not available
Saharman et al. [5]	Indonesia	April–October 2013 and April–August 2014	Patients and HCW	Throat swabs and rectal swabs or stool (patients), and throat and rectal swabs (HCW)	Yes, 5 mL of TSB with 2 mg/L cefotaxime and 50 mg/L vancomycin	No	12.4%^b^
Torrens et al. [51]	Bulgaria, Czech Republic, Spain, Netherlands, Serbia, Germany, Estonia, Hungary, UK, Turkey, and France	2016–2021	Patients	Perianal swabs	No	Yes, CHROMID *P. aeruginosa* agar (bioMérieux, France)	46.5%
Wendel et al. [34]	Germany	January 2015–June 2020	Patients	Rectal swabs, nose swabs, throat swabs	No	No	Not available

*CR-PA* carbapenem-resistant *Pseudomonas aeruginosa*, *HCW* healthcare workers, *SDD* selective digestive tract decontamination, *TSB* tryptic soy broth, *VIM-PA* Verona Integron-encoded Metallo-beta-lactamase (VIM)-producing *Pseudomonas aeruginosa*

^a^*P. aeruginosa* isolates were only tested for the presence of the VIM β-lactamase enzyme

^b^Prevalence unavailable for HCW

**Fig. 2:**
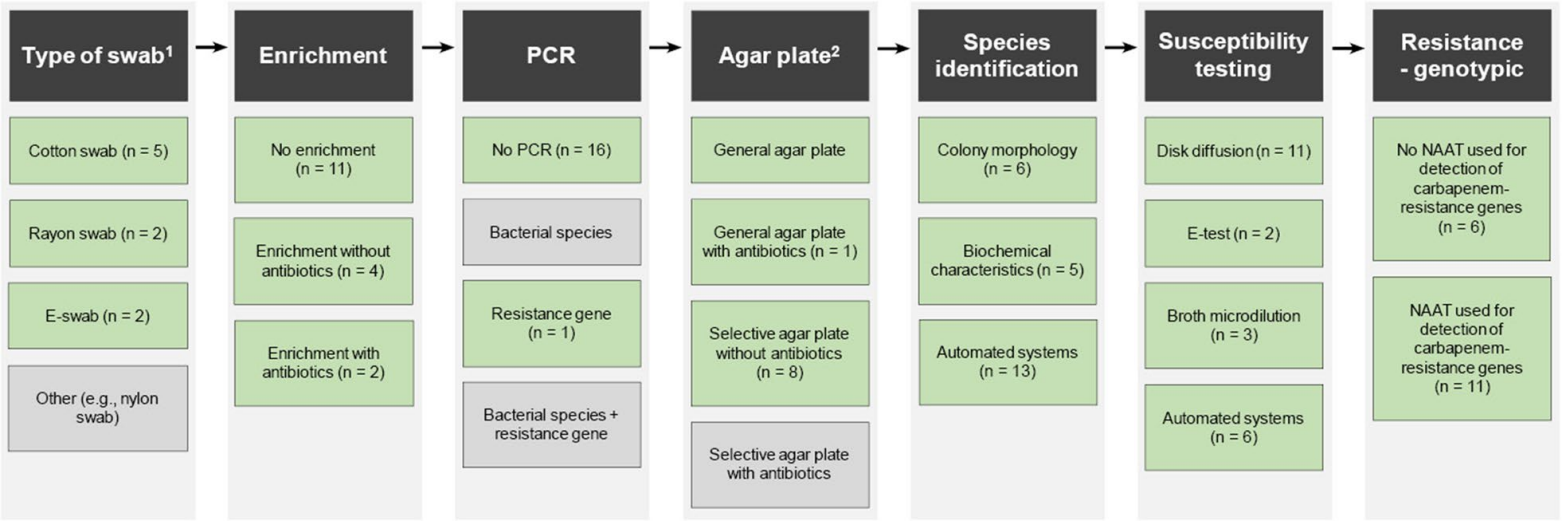
Overview of steps to identify CR-PA carriage in outbreak-surveillance studies with surveillance samples (n = 17). *NAAT* nucleic acid amplification test. Green color indicates that the method is reported in outbreak-surveillance studies with surveillance samples; grey color indicates not being reported.^1^ Missing or not applicable in 8 articles. ^2^ Three articles described the use of multiple agar plates. This figure summarizes the methods used in the included outbreak-surveillance studies with surveillance samples

Regarding AST, Kirby-Bauer disk diffusion (n = 11, 65%) was most commonly used, followed by automated systems (n = 6, 35%), broth microdilution (n = 3, 18%), and E-test (n = 2, 12%). Automated systems included the VITEK (n = 5), BD-Phoenix (n = 1), and Sensititre (ThermoFisher) (n = 1) [16, 17, 29, 32–34]. A variety of additional culture-based methods for carbapenemase detection were used in 10 studies (59%), with the double-disk synergy test (DDST) (n = 4) most commonly described. Lastly, nucleic acid amplification tests (NAATs) to detect carbapenemase genes were used in 11 studies (65%). In summary, Fig. 2 presents all the different steps taken in outbreak-surveillance studies with surveillance cultures. The culture methods used per included study are available in Table S4.

#### Study quality

The methodological quality of fifteen studies was assessed with the STROBE guideline. Thirteen studies were of medium quality (scores between 12 and 22 points), and two studies of high quality, scoring 23 and 24 points. Two outbreak studies were assessed as being of medium quality (scoring 24 and 26 points, respectively) following the ORION guideline.

### Outbreak-surveillance studies with clinical samples

In total, 116 outbreak-surveillance studies with clinical samples were included. Table S5 lists the used culture methods. In short, the majority of studies (n = 67, 57.8%) did not mention the type of swab. Of the thirty-six studies (31.0%) that did, cotton swabs were mostly used (n = 28, 77.8%). An enrichment step was reported in 9 studies (7.8%), and included a variety of broths. Brain heart infusion broth (n = 2, 22%), TSB (n = 2, 22%), and thioglycolate broth (n = 2, 22%) were most commonly reported. One-quarter of studies used selective media, including *Pseudomonas* Cetrimide agar (n = 24, 80%), CHROMO agar (n = 1, 3%), ChromID OXA-48 (n = 1, 3%), CHROMagar KPC (n = 1, 3%), CHROMID *P. aeruginosa* (n = 1, 3%), Pseudomonas agar (n = 1, 3%), and MacConkey agar supplemented with 1 µg/mL meropenem (n = 1, 3%). A variety of additional phenotypic methods for carbapenemase detection were described in 50 studies (43.1%), and included the Modified Hodge Test (MHT) (n = 8, 16%), combined-disk test (n = 7, 14%), and modified Carbapenem Inactivation Method (mCIM) (n = 7, 14%). Lastly, 42 studies (36.2%) reported using NAATs for the detection of carbapenemase genes.

## Discussion

This systematic review aimed to determine the most sensitive culture method and summarizes the culture methods used to detect CR-PA in humans. The limited number of diagnostic accuracy studies identified a significant benefit of using an enrichment broth prior to culturing the material on a selective agar plate and found two selective agar plates with 100% sensitivity for the detection of CR-PA. Recent outbreak-surveillance studies described the use of a large variety of products and culture methods to detect both carriage and infection. In the next paragraphs, we provide an overview of the findings related to each step within the process to detect CR-PA in humans (Fig. 2).

### Sampling sites used for surveillance

Perianal swabs, rectal swabs or stools were most often used to screen patients in outbreak-surveillance studies with surveillance samples, as it is recommended by (inter)national guidelines for highly resistant microorganisms or multidrug-resistant Gram-negative bacteria [35, 36]. Whether these body sites are most optimal for the detection of CR-PA could not be determined because there were no studies available comparing the yield of sampling different body sites for CR-PA. Interestingly, Warnke et al. showed that for *P. aeruginosa* (regardless of susceptibility), yields may be higher with rectal swabbing than with perianal swabbing in hospitalized patients [37].

### Type of swab

For surveillance samples, where the expected bacterial burden is lower compared to an infection, the type of swab used may be critical. The total number of studies, both diagnostic accuracy and outbreak-surveillance studies, reporting on the type of swab used to collect samples was limited. Overall, the cotton swab was most commonly mentioned, possibly due to the fact that cotton swabs are cheap and widely available. Interestingly, cotton swabs showed comparable detection rates to flocked swabs when used in rectal screening for multidrug-resistant bacteria, suggesting cotton swabs could indeed be a suitable swab for collecting surveillance samples to determine CR-PA carriage [38]. Yet, another study found superior performance of polyurethane-cellular-foam and nylon-flocked swabs compared to rayon swabs for the recovery of Gram-negative bacteria, including *P. aeruginosa* [37]. However, the latter study did not include cotton swabs in the comparison and only investigated the recovery rates from perianal and rectal screening sites. The limited amount of data provided by included studies prevents us from drawing conclusions on which type of swab provides the highest yield for the detection of CR-PA.

### Culture methods

The use of an enrichment broth, whether selective or non-selective, prior to inoculation on a selective medium was found to significantly increase the detection of CR-PA [27]. Similarly, enrichment of (surveillance) swabs using a broth also increased the detection of other multidrug-resistant microorganisms, such as methicillin-resistant *Staphylococcus aureus* and extended-spectrum-beta-lactamase-producing bacteria [39, 40]. In general, smaller populations of bacteria are likely to be detected with the enrichment broth, increasing sensitivity. A broad array of enrichment broths and selective media were described in outbreak-surveillance studies. Based on this observed variety in practice, consensus on a particular type of enrichment broth, selective medium, and the most sensitive combination thereof appears to be lacking. Possible explanations could be the lack of international guidance on the methods used to detect CR-PA carriage in humans as well as differences in product availability around the world. The variety of media around the globe may nevertheless be of benefit when supply chain disruptions occur.

In diagnostic accuracy studies, two different selective media, chromID CARBA and HardyCHROM CRE, were both found to have 100% sensitivity for the detection of CR-PA. When combining this measure of test accuracy with available information on storage requirements and product availability, neither of these plates can be recommended as these were not available worldwide. Also, both plates were investigated in one study only involving a limited amount of CR-PA strains, thereby, some caution is needed in interpreting these findings.

Although our search was not aimed at identifying methods used to detect carbapenemases, we did collect such information. The wide variety of phenotypic methods and the methods’ descriptions showed that international consensus and guidance is also lacking for this.

### TAT

Although the use of an enrichment broth was found to significantly increase the detection of CR-PA, it extended the TAT by 18–24 h. Selective media, on the other hand, were found to reduce the TAT by a day as opposed to using routine culture media. Interestingly, the isolation of carbapenem-resistant Enterobacterales from rectal surveillance swabs was improved by extending the incubation time on the plate (overall more than 24 h), which mostly enabled the detection of less resistant strains [41]. Based on our findings, the combination of an enrichment broth and selective medium seems to be the most sensitive method for the detection of CR-PA with a shortest possible TAT of two days. Furthermore, methods combining several steps, such as species identification directly followed by methods for carbapenemase detection, have the potential to reduce the TAT without significantly compromising test accuracy. These reductions in TAT can have major implications for patient outcomes, since effective antimicrobial therapy is crucial in critically ill patients and a timely switch to optimal antimicrobial therapy is supporting antibiotic stewardship efforts.

### Limitations

This systematic review has certain limitations. First, we received a limited number of replies from corresponding authors to our requests for approval and provision of missing data. A possible explanation could be the timing of our requests, which was amidst the COVID-19 pandemic. Second, to determine which culture method is most feasible to be implemented in the clinical setting, product availability data was collected from the manufacturer’s website and cross-checked with local data from Indonesia and the Netherlands. Product availability, however, may vary worldwide.

### Implications for future research

Several implications for future research can be derived from this comprehensive review. First, it remains unknown which body site is most optimal to identify CR-PA carriage. Second, more elaborate research is needed to determine the most sensitive culture method to detect CR-PA. Studies investigating and comparing the use of different enrichment broths and selective media are currently limited and lacking with regard to the best combination of enrichment broth and plate. Also, when carbapenem antibiotics are added to broths or plates in the included studies, either meropenem or imipenem is used. Other (carbapenem) antibiotics should be considered as well. Further, it is important that a wide variety of CR-PA are included in such studies (e.g., including not only VIM-positive but also IMP-, GES-, and NDM-positive *P. aeruginosa* strains, as well as non-carbapenemase producing high-risk clones), as many current studies are often skewed towards only including CR-PA isolates according to local epidemiology. Nevertheless, although the general view is that a screening method for CR-PA (or any multidrug-resistant pathogen) should be as sensitive as possible, especially in outbreak settings, it should be noted that the number of bacteria needed for transmission of CR-PA from a CR-PA-positive patient to a previously negative source or patient is unknown. Third, efforts aimed at reducing the TAT without compromising test accuracy remain of clinical importance. A direct PCR on the broth to determine the presence of carbapenemase genes could be further investigated and compared to more commonly applied approaches in terms of its effect on the TAT. However, PCR has significant resource implications, and may therefore not be feasible in all settings. Fourth, there is an imbalance in recent outbreak-surveillance studies, with the majority of studies reporting on clinical compared to surveillance samples. Moreover, the latter studies provide only limited information with regard to surveillance among healthy humans and HCW. This was also recognized in a recently published systematic review by Büchler et al. and could be caused by underreporting of surveillance or the fact that surveillance is actually not performed [42]. Future research focusing on surveillance is important in order to get insight into the worldwide dissemination of high-risk clones and modes of transmission of CR-PA. International guidance on screening methods for CR-PA can be of added value for this.

## Conclusions

In conclusion, we found some evidence of a significant benefit of using an enrichment broth prior to plating the material on a selective medium for the detection of CR-PA. Furthermore, the scarcity of diagnostic accuracy studies comparing different culture methods and the large variety of culture methods described in recent outbreak-surveillance studies reflects a lack of knowledge on the methods to be used for the rapid and sensitive detection of CR-PA. Future research is needed to determine which sampling site and culture methods, including broths and media, are most sensitive for the detection of CR-PA.

### Supplementary Information

Supplementary Material 1. File S1. Search strategy.

Supplementary Material 2. Table S1. PRISMA 2020 checklist.

Supplementary Material 3. Table S2. Quality assessment scores of included diagnostic accuracy studies and outbreak-surveillance studies with surveillance samples.

Supplementary Material 4. Table S3. Product information from products used in diagnostic accuracy studies.

Supplementary Material 5. Table S4. Culture methods and numbers of samples reported in outbreak-surveillance studies with surveillance samples (n = 17).

Supplementary Material 6. Table S5. Culture methods reported in outbreak-surveillance studies with clinical samples (n = 116).

## Data Availability

All data generated or analysed during this study are included in this published article and its supplementary information files.

## Abbreviations

AST: Antimicrobial susceptibility test
CR-PA: Carbapenem-resistant *Pseudomonas aeruginosa*
DSST: Direct sputum antimicrobial susceptibility testing
HCW: Healthcare worker
mCIM: modified Carbapenem Inactivation Method
MHT: Modified Hodge Test
NAAT: Nucleic acid amplification test
ORION: Outbreak Reports and Intervention studies Of Nosocomial infection
PRISMA: Preferred Reporting Items for Systematic Reviews and Meta-Analyses
QUADAS-2: Quality Assessment of Diagnostic Accuracy Studies
STROBE: Strengthening Reporting of Observational Studies in Epidemiology
TAT: Turnaround time
WHO: World Health Organization
