# Supplementary material for: In search of the best method to detect carriage of carbapenem-resistant Pseudomonas aeruginosa in humans: a systematic review
Source: Ann Clin Microbiol Antimicrob. 2024 Jun 10;23:50. doi: 10.1186/s12941-024-00707-1 (PMC11163693; doi:10.1186/s12941-024-00707-1)
Supplement: Supplementary file 1 — Supplementary Material 1. File S1. Search strategy. [file 12941_2024_707_MOESM1_ESM.docx]

**SUPPLEMENTARY FILE S1: SEARCH STRATEGY**

| **Database searched** | **Platform** | **Years of coverage** | **Records** | **Records after duplicates removed** |
| --- | --- | --- | --- | --- |
| Embase | Embase.com | 1971 - Present | 5793 | 5725 |
| Medline ALL | Ovid | 1946 - Present | 3411 | 957 |
| Web of Science Core Collection* | Web of Knowledge | 1975 - Present | 2643 | 486 |
| Cochrane Central Register of Controlled Trials** | Wiley | 1992 - Present | 23 | 11 |
| **Total** | | | **11870** | **7179** |

*Science Citation Index Expanded (1975-present) ; Social Sciences Citation Index (1975-present) ; Arts & Humanities Citation Index (1975-present) ; Conference Proceedings Citation Index- Science (1990-present) ; Conference Proceedings Citation Index- Social Science & Humanities (1990-present) ; Emerging Sources Citation Index (2005-present)

** Manually deleted abstracts from trial registries

No other database limits were used than those specified in the search strategies

**Embase.com**

('Pseudomonas aeruginosa'/exp/mj OR 'Pseudomonas infection'/mj OR Pseudomonas/mj OR (Pseudomonas* OR P-aeruginosa*):ti) AND ('multidrug resistance'/de OR 'drug resistance'/de OR 'antibiotic resistance'/exp OR 'antibiotic sensitivity'/de OR 'extensive drug resistance'/de OR 'carbapenem resistant Pseudomonas aeruginosa'/de OR 'multidrug resistant Pseudomonas aeruginosa'/de OR ((resistan* OR sensitiv*) NEAR/3 (multidrug* OR drug* OR imipenem* OR meropenem* OR carbapenem* OR beta-lactam* OR antibiot* OR anti-biot* OR antimicrob* OR anti-microb*)):ab,ti) AND ('culture medium'/exp OR 'agar'/de OR 'cell culture technique'/de OR 'enrichment culture'/de OR 'microbiological examination'/de OR 'bacterium examination'/de OR ('comparative study'/de AND 'diagnosis'/exp) OR 'diagnostic accuracy'/de OR 'diagnostic test accuracy study'/de OR 'broth dilution'/de OR 'intermethod comparison'/de OR (Culture-Medi* OR Agar* OR Broth* OR Differential-Medi* OR Selective-Medi* OR Test-Medi* OR Defined-Medi* OR Culture-Technique* OR Microbiological-Technique* OR Microbiological-examinat* OR bacter*-examinat* OR Testing-Method* OR enrichment-culture* OR medium OR (comparative-stud* AND diagnos*) OR ((intermethod* OR method*) NEAR/3 compar*) OR (diagnostic* NEAR/3 accura*)):ab,ti OR (media OR culture*):ti)

('Pseudomonas aeruginosa'/exp/mj OR 'Pseudomonas infection'/mj OR Pseudomonas/mj OR (Pseudomonas* OR P-aeruginosa*):ti) AND ('contact examination'/exp OR 'outbreak'/de OR 'disease transmission'/de OR 'bacterial transmission'/de OR 'epidemic'/de OR 'endemic disease'/de OR 'hospital infection'/de OR 'cross infection'/de OR ((contact* NEAR/3 (detect* OR examinat* OR trac* OR search*)) OR outbreak* OR transmission* OR epidemic* OR Nosocomial* OR endemic* OR ((hospital* OR cross) NEAR/3 infection*)):ab,ti) AND ('multidrug resistance'/de OR 'drug resistance'/de OR 'antibiotic resistance'/exp OR 'antibiotic sensitivity'/de OR 'extensive drug resistance'/de OR 'carbapenem resistant Pseudomonas aeruginosa'/de OR 'multidrug resistant Pseudomonas aeruginosa'/de OR ((resistan* OR sensitiv*) NEAR/3 (multidrug* OR multi-drug* OR imipenem* OR meropenem* OR carbapenem* OR beta-lactam* OR antibiot* OR anti-biot* OR antimicrob* OR anti-microb*)):ab,ti)

**Medline ALL Ovid**

(*Pseudomonas aeruginosa/ OR *Pseudomonas Infections/ OR *Pseudomonas/ OR (Pseudomonas* OR P-aeruginosa*).ti.) AND (Drug Drug Resistance/ OR Drug Resistance, Microbial/ OR Drug Resistance, Multiple, Bacterial/ OR ((resistan* OR sensitiv*) ADJ3 (multidrug* OR multi-drug* OR imipenem* OR meropenem* OR carbapenem* OR beta-lactam* OR antibiot* OR anti-biot* OR antimicrob* OR anti-microb*)).ab,ti.) AND (exp Culture Media/ OR Agar/ OR exp Cell Culture Techniques/ OR (Comparative Study/ AND exp Diagnosis/) OR (Culture-Medi* OR Agar* OR Broth* OR Differential-Medi* OR Selective-Medi* OR Test-Medi* OR Defined-Medi* OR Culture-Technique* OR Microbiological-Technique* OR Microbiological-examinat* OR bacter*-examinat* OR Testing-Method* OR enrichment-culture* OR medium OR (comparative-stud* AND diagnos*) OR ((intermethod* OR method*) ADJ3 compar*) OR (diagnostic* ADJ3 accura*)).ab,ti. OR (media OR culture*).ti.)

(*Pseudomonas aeruginosa/ OR *Pseudomonas Infections/ OR *Pseudomonas/ OR (Pseudomonas* OR P-aeruginosa*).ti.) AND (Contact Tracing/ OR Disease Outbreaks/ OR exp Disease Transmission, Infectious/ OR Epidemics/ OR Endemic Diseases/ OR Cross Infection/ OR ((contact* ADJ3 (detect* OR examinat* OR trac* OR search*)) OR outbreak* OR transmission* OR epidemic* OR Nosocomial* OR endemic* OR ((hospital* OR cross) ADJ3 infection*)).ab,ti.) AND (Drug Drug Resistance/ OR Drug Resistance, Microbial/ OR Drug Resistance, Multiple, Bacterial/ OR ((resistan* OR sensitiv*) ADJ3 (multidrug* OR multi-drug* OR imipenem* OR meropenem* OR carbapenem* OR beta-lactam* OR antibiot* OR anti-biot* OR antimicrob* OR anti-microb*)).ab,ti.)

**Web of Science Core Collection**

((TI=(Pseudomonas* OR P-aeruginosa*)) AND TS=(((resistan* OR sensitiv*) NEAR/2 (multidrug* OR drug* OR imipenem* OR meropenem* OR carbapenem* OR beta-lactam* OR antibiot* OR anti-biot* OR antimicrob* OR anti-microb*))) AND (TS=(Culture-Medi* OR Agar* OR Broth* OR Differential-Medi* OR Selective-Medi* OR Test-Medi* OR Defined-Medi* OR Culture-Technique* OR Microbiological-Technique* OR Microbiological-examinat* OR bacter*-examinat* OR Testing-Method* OR enrichment-culture* OR medium OR (comparative-stud* AND diagnos*) OR ((intermethod* OR method*) NEAR/2 compar*) OR (diagnostic* NEAR/2 accura*)) OR TI=(media OR culture*)))

TI=((Pseudomonas* OR P-aeruginosa*)) AND TS=((((contact* NEAR/2 (detect* OR examinat* OR trac* OR search*)) OR outbreak* OR transmission* OR epidemic* OR Nosocomial* OR endemic* OR ((hospital* OR cross) NEAR/2 infection*))) AND (((resistan* OR sensitiv*) NEAR/2 (multidrug* OR multi-drug* OR imipenem* OR meropenem* OR carbapenem* OR beta-lactam* OR antibiot* OR anti-biot* OR antimicrob* OR anti-microb*))))

**Cochrane CENTRAL register of Trials**

((Pseudomonas* OR P NEXT aeruginosa*):ti) AND (((resistan* OR sensitiv*) NEAR/3 (multidrug* OR drug* OR imipenem* OR meropenem* OR carbapenem* OR beta NEXT lactam* OR antibiot* OR anti NEXT biot* OR antimicrob* OR anti NEXT microb*)):ab,ti) AND ((Culture NEXT Medi* OR Agar* OR Broth* OR Differential NEXT Medi* OR Selective NEXT Medi* OR Test NEXT Medi* OR Defined NEXT Medi* OR Culture NEXT Technique* OR Microbiological NEXT Technique* OR Microbiological NEXT examinat* OR bacter* NEXT examinat* OR Testing NEXT Method* OR enrichment NEXT culture* OR medium OR (comparative NEXT stud* AND diagnos*) OR ((intermethod* OR method*) NEAR/3 compar*) OR (diagnostic* NEAR/3 accura*)):ab,ti OR (media OR culture*):ti)

((Pseudomonas* OR P-aeruginosa*):ti) AND (((contact* NEAR/3 (detect* OR examinat* OR trac* OR search*)) OR outbreak* OR transmission* OR epidemic* OR Nosocomial* OR endemic* OR ((hospital* OR cross) NEAR/3 infection*)):ab,ti) AND (((resistan* OR sensitiv*) NEAR/3 (multidrug* OR multi-drug* OR imipenem* OR meropenem* OR carbapenem* OR beta-lactam* OR antibiot* OR anti-biot* OR antimicrob* OR anti-microb*)):ab,ti)
