# Supplementary material for: In search of the best method to detect carriage of carbapenem-resistant Pseudomonas aeruginosa in humans: a systematic review
Source: Ann Clin Microbiol Antimicrob. 2024 Jun 10;23:50. doi: 10.1186/s12941-024-00707-1 (PMC11163693; doi:10.1186/s12941-024-00707-1)
Supplement: Supplementary file 3 — Supplementary Material 3. Table S2. Quality assessment scores of included diagnostic accuracy studies and outbreak-surveillance studies with surveillance samples. [file 12941_2024_707_MOESM3_ESM.docx]

**SUPPLEMENTARY TABLE S2: QUALITY ASSESSMENT SCORES OF INCLUDED DIAGNOSTIC ACCURACY AND OUTBREAK-SURVEILLANCE STUDIES WITH SURVEILLANCE SAMPLES.**

| Quality Threshold Scores | | QUADAS-2 [1] (N=6) | STROBE [2] (N=15) | ORION [3] (N=2) |
| --- | --- | --- | --- | --- |
| Low (%) | QUADAS-2 0-6 | 0 (0.0) |  |  |
|  | STROBE 0-11 |  | 0 (0.0) |  |
|  | ORION 0-17 |  |  | 0 (0.0) |
| Medium (%) | QUADAS-2 7-12  STROBE 12-22  ORION 18-34 | 1 (16.7) | 13 (86.7) | 2 (100) |
| High (%) | QUADAS-2 13-18  STROBE 23-33  ORION 35-52 | 5 (83.3) | 2 (13.3) | 0 (0.0) |

1. Whiting PF, Rutjes AW, Westwood ME, Mallett S, Deeks JJ, Reitsma JB, et al. QUADAS-2: a revised tool for the quality assessment of diagnostic accuracy studies. Ann Intern Med. 2011;155(8):529-36. <https://doi.org/10.7326/0003-4819-155-8-201110180-00009>.

2. von Elm E, Altman DG, Egger M, Pocock SJ, Gøtzsche PC, Vandenbroucke JP, et al. Strengthening the Reporting of Observational Studies in Epidemiology (STROBE) statement: guidelines for reporting observational studies. Bmj. 2007;335(7624):806-8. <https://doi.org/10.1016/j.ijsu.2014.07.013>.

3. Stone SP, Cooper BS, Kibbler CC, Cookson BD, Roberts JA, Medley GF, et al. The ORION statement: guidelines for transparent reporting of outbreak reports and intervention studies of nosocomial infection. Lancet Infect Dis. 2007;7(4):282-8. <https://doi.org/10.1016/s1473-3099(07)70082-8>.
