# Supplementary material for: In search of the best method to detect carriage of carbapenem-resistant Pseudomonas aeruginosa in humans: a systematic review
Source: Ann Clin Microbiol Antimicrob. 2024 Jun 10;23:50. doi: 10.1186/s12941-024-00707-1 (PMC11163693; doi:10.1186/s12941-024-00707-1)
Supplement: Supplementary file 4 — Supplementary Material 4. Table S3. Product information from products used in diagnostic accuracy studies. [file 12941_2024_707_MOESM4_ESM.docx]

**SUPPLEMENTARY TABLE S3: PRODUCT INFORMATION FROM PRODUCTS USED IN DIAGNOSTIC ACCURACY STUDIES.**

| **Product name** | **Brand** | **Storage** | **Availability**^a^ | **Incubation time as indicated by the supplier** | **Product picture** |
| --- | --- | --- | --- | --- | --- |
| *Enrichment broth* | | | | | |
| Tryptic soy broth | Sigma-Aldrich, Buchs, Switzerland | Available as dehydrated powder, store at 2-25˚C in a dry place. | High | Not applicable | 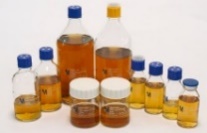 |
| *Selective media* | | | | | |
| Cetrimide agar | Oxoid, Basingstoke, UK | Available as dehydrated medium, store at 10-30˚C. | Not widely available^b^ | Up to 48 hours | 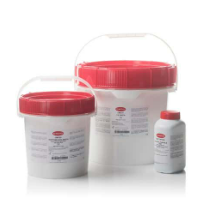 |
|  | bioMérieux, Marcy L'Étoile, France | Available as poured plates. | Not widely available | Information not available | 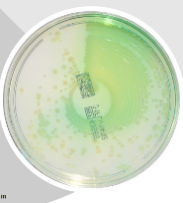 |
| CHROMagar *Pseudomonas* | CHROMagarᵀᴹ | Available as dehydrated powder, store at 15-30˚C in a dry place away from light. | Available | 24/26 hours, extension to 48 hours for fragile *Pseudomonas* species (small colonies, etc.) | 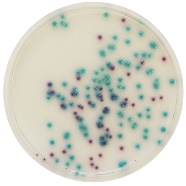 |
| *Pseudomonas* C-N selective agar | Oxoid, Basingstoke, UK | Available as poured plates, store at 2-12˚C. | Not widely available^b^ | Information not available | 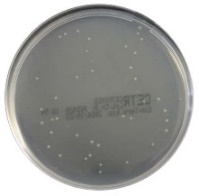 |
| *Pseudomonas*  chromogenic medium | bioMérieux, La Balme-les-Grottes, France | Available as poured plates. | Not widely available | 24 hours | 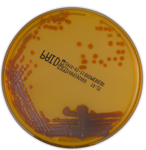 |
| HardyCHROM CRE agar | Hardy Diagnostics | Available as poured plates. | Not widely available | 18-24 hours | 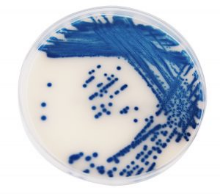 |
| chromID CARBA agar | bioMérieux | Available as poured plates. | Not widely available | 18 hours | 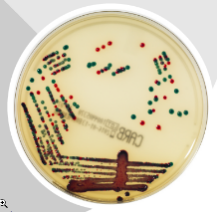 |
| *Supplement* | | | | | |
| Meropenem | Sigma-Aldrich, Buchs, Switzerland | Available as powder | Available | Not applicable | Not available |

^a^ Availability determined from the manufacturers’ websites. Subsequently, it was cross-checked if it could be ordered in the Netherlands and Indonesia as proof of worldwide availability (last checked in 2023).

^b^ Not available in Indonesia
