# Supplementary material for: In search of the best method to detect carriage of carbapenem-resistant Pseudomonas aeruginosa in humans: a systematic review
Source: Ann Clin Microbiol Antimicrob. 2024 Jun 10;23:50. doi: 10.1186/s12941-024-00707-1 (PMC11163693; doi:10.1186/s12941-024-00707-1)
Supplement: Supplementary file 5 — Supplementary Material 5. Table S4. Culture methods and numbers of samples reported in outbreak-surveillance studies with surveillance samples (n = 17). [file 12941_2024_707_MOESM5_ESM.docx]

**SUPPLEMENTARY TABLE S4: CULTURE METHODS AND NUMBERS OF SAMPLES REPORTED IN OUTBREAK-SURVEILLANCE STUDIES WITH SURVEILLANCE SAMPLES (N=17)**

**Table 2a** Identification methods reported in outbreak-surveillance studies with surveillance samples (n=17).

| Reference | Type of swab used | Methods used for identification | | | |  |
| --- | --- | --- | --- | --- | --- | --- |
|  |  | **Colony morphology** | **Biochemical characteristics** | **Automated system** | **PCR for direct species identification** | **PCR for species confirmation** |
| Adelantado et al. [1] | Not available | No | No | Yes: MALDI-TOF MS | No | No |
| Catho et al. [2] | E-swab | No | No | Yes: MALDI-TOF MS | No | No |
| DeGeyter et al. [3] | E-swab | No | No | Yes: MALDI-TOF MS | No | No |
| Franco et al. [4] | Cotton swab | Yes | Yes | Yes: VITEK-2 | No | No |
| Freire et al. [5] | Rayon swab with a semisolid transport medium. | No | No | Yes: VITEK or MALDI-TOF | Yes | No |
| Hu et al. [6] | N/A | No | No | Yes: MALDI-TOF MS | No | No |
| Karampatakis et al. [7] | Cotton swab | No | No | Yes: VITEK-2 | No | No |
| Maclean et al. [8] | Cotton swab | Yes | Yes | No | No | Yes: targeting 16S rDNA gene. |
| Mahmoud et al. [9] | Cotton swab | Yes | Yes | No | No | No |
| Martak et al. [10] | N/A; not available | No | No | Yes: MALDI-TOF MS | No | No |
| Ohadian Moghadam et al. [11] | N/A | Yes | Yes | No | No | Yes: targeting *oprL* gene. |
| Odoi et al. [12] | Not available | Yes | Yes | No | No | Yes: targeting *oprL* gene. |
| Pham et al. [13] | Not available | Yes | No | Yes: prior to 2013 by VITEK-2, after 2013 by MALDI-TOF. | No, not for species identification, however, a direct PCR was done on the broth for *bla_VIM_* detection. | No |
| Rice et al. [14] | Not available | No | No | Yes: MALDI-TOF MS | No | No |
| Saharman et al. [15] | Sterile cotton-tipped swabs | No | No | Yes: VITEK-2 & MALDI Biotyper | No | No |
| Torrens et al. [16] | Not available | No | No | Yes: MALDI-TOF MS | No | No |
| Wendel et al. [17] | Rayon swab | No | No | Yes: VITEK-2 or MALDI-TOF. | No | No |

*MALDI-TOF* Matrix-Assisted Laser Desorption/Ionization Time-Of-Flight mass spectrometry, *N/A* not applicable, *SDD* selective digestive tract decontamination, *TSB* tryptic soy broth, *VIM-PA* Verona Integron-encoded Metallo-beta-lactamase (VIM) producing *Pseudomonas aeruginosa.*

**Table 2b** Methods for susceptibility testing and detection of carbapenemases reported in outbreak-surveillance studies with surveillance samples (n=17).

| Reference | Method used for susceptibility test | | | | | Additional culture method used for carbapenemase detection | NAAT used for detection of carbapenemase genes. |
| --- | --- | --- | --- | --- | --- | --- | --- |
|  | **(Kirby-Bauer) disk diffusion** | **E-test** | **Broth microdilution** | **Agar dilution method** | **Automated system** |  |  |
| Adelantado et al. [1] | Yes (EUCAST) | No | No | No | No | No | Yes |
| Catho et al. [2] | Yes (EUCAST) | No | No | No | Yes: Sensititre (ThermoFisher) | No | Yes |
| DeGeyter et al. [3] | No | No | No | No | No | No | No |
| Franco et al. [4] | No | No | No | No | Yes: VITEK-2 | Yes, inhibition with EDTA-carbapenem inhibition method using disks. | Yes |
| Freire et al. [5] | No | No | No | No | Yes: VITEK (CLSI) | Yes, imipenem-cloxacillin combined disk method. | Yes |
| Hu et al. [6] | Yes | No | Yes (CLSI), on all CR-PA | No | No | Yes, sCIM. | Yes |
| Karampatakis et al. [7] | Yes (CLSI) | Yes | No | No | Yes: VITEK-2 | Yes, however, the method is not mentioned. | Yes |
| Maclean et al. [8] | Yes (CLSI) | No | No | No | No | Yes, imipenem-EDTA DDST. | No |
| Mahmoud et al. [9] | Yes (CLSI) | No | No | No | No | No | No |
| Martak et al. [10] | Not available | Not available | Not available | Not available | Not available | No | No |
| Ohadian Moghadam et al. [11] | Yes (CLSI) | Yes (CLSI) | No | No | No | Yes, a carbapenemase assay was perfomed with modified Hodge Test and imipenem-EDTA DDST | Yes, on all carbapenem-resistant strains. |
| Odoi et al. [12] | Yes (EUCAST) | No | No | No | No | Yes, imipenem-EDTA synergy test and phenylboronic acid test. | Yes |
| Pham et al. [13] | No | No | No | No | Yes: VITEK-2 (EUCAST) | No | Yes |
| Rice et al. [14] | Yes (EUCAST) | No | No | No | No | Yes, NG test CARBA 5 (Hardy Diagnostics). | No |
| Saharman et al. [15] | Yes (EUCAST) | No | No | No | No (only for clinical isolates) | Yes, imipenem/doripenem combination disk test with EDTA | Yes |
| Torrens et al. [16] | No | No | Yes (EUCAST) | No | No | Yes, DDST. | Yes |
| Wendel et al. [17] | Yes (initial AST) | No | Yes (Micronaut-S *Pseudomonas* MIC panels for confirmation of initial AST results). | No | Yes: VITEK-2 or BD-Phoenix (initial AST). | No | No |

*AST* antimicrobial susceptibility test, *CLSI* Clinical & Laboratory Standards Institute, *CR-PA* carbapenem-resistant *Pseudomonas aeruginosa*, *DDST* double-disk synergy test, *EDTA* ethylenediaminetetraacetic acid, *EUCAST* European Committee of Antimicrobial Susceptibility Testing, *sCIM* simplified Carbapenem Inactivation Method.

**Table 2c** Numbers of samples reported in outbreak-surveillance studies with surveillance samples (n=17).

| Reference | Country | Study period | Sampling sites used for surveillance samples | N of samples overall | N of patients/persons sampled | N of samples with CR-PA | N of patients/persons with CR-PA | Prevalence (n of persons with CR-PA/n of persons sampled) |
| --- | --- | --- | --- | --- | --- | --- | --- | --- |
| Adelantado et al. [1] | Spain | January - December 2019 | Perianal swabs | 1867 swabs of which 255 *P. aeruginosa* cases were identified | Not available | 64 | Not available | Not available |
| Catho et al. [2] | Switzerland | March 2018 - September 2020 | Perianal swabs | Not available | Not available | Not available | 10 | Not available |
| DeGeyter et al. [3] | Belgium | January - December 2019 and October - December 2020 | Rectal swabs | Not available | Not available | 0^1^ | 0^1^ | 0%^1^ |
| Franco et al. [4] | Paraguay | November 2009 - December 2015 | Rectal swabs | Not available | Not available | Not available | Not available | Not available |
| Freire et al. [5] | Brazil | February 2019 - February 2020 | Rectal swabs | 905 paired samples | 399 | 22 paired samples | 12 with RT-PCR and surveillance culture positive, 5 only RT-PCR positive, and 4 only surveillance culture positive. | 5.3% |
| Hu et al. [6] | China | January 2014 - December 2019 | Fecal samples | 4560 of which 184 were identified as *P. aeruginosa.* | 4560 | 76 | 76 | 1.7% |
| Karampatakis et al. [7] | Greece | August 2012 - November 2016 and December 2016 - December 2017 | Rectal swabs | 3126 | 1226 | 34 | 34 | 2.8% |
| Maclean et al. [8] | South Africa | June 2018 - June 2019 | Ear swabs | 47 of which 2 were identified as *P. aeruginosa.* | 28 | 2 | 2 | 7.1% |
| Mahmoud et al. | Egypt | December 2017 – March 2020 | Hands (HCW) | 30 of which 3 were identified as *P. aeruginosa* | Not available | 0 | Not available | Not available |
| Martak et al. [10] | France & Germany | November 2017 - April 2019 | Fecal samples | Not available | 403 of which 208 were *P. aeruginosa* carriers. | 12 | 12 | 3.0% |
| Ohadian Moghadam et al. [11] | Iran | January 2018 - January 2020 | Urine | 157 of which 68 were identified as *P. aeruginosa.* | 157 | 38 | 38 | 24.2% |
| Odoi et al. [12] | Ghana | September 2015 - July 2016 | Stool and urine (patients), and hand swabs (farmers). | 264 of which 47 were identified as *P. aeruginosa* | 264 of which 78 from stool, 97 from urine, and 89 hand swabs. | 8 | 8 | 3.0% |
| Pham et al. [13] | The Netherlands | January 1, 2010 - May 18, 2018. | Throat swabs and rectal swabs. | Not available | 7549 | Not available | 62^1^ | 0.8%^1^ |
| Rice et al. [14] | England | September 2016 - November 2020 | Throat swabs, rectal swabs, and wounds (including line sites). | Not available | Not available | 4 (rectal swabs) | 4 | Not available |
| Saharman et al. [15] | Indonesia | April - October 2013 and April - August 2014 | Throat swabs and rectal swabs or stool (patients), and throat and rectal swabs (HCW). | 2137 (1067 throat swabs and 1070 rectal swabs or stool)^2^ | 412^2^ | 82 (39 throat swabs and 43 rectal swabs or stool) ^2^ | 51 (8 throat swabs only, 12 rectal swab only, and 31 both) ^2^ | 12.4%^2^ |
| Torrens et al. [16] | Bulgaria, Czech Republic, Spain, the Netherlands, Serbia, Germany, Estonia, Hungary, United Kingdom, Turkey, and France. | 2016 - 2021 | Perianal swabs | 541 | 297 | 138 | 138 | 46.5% |
| Wendel et al. [17] | Germany | 2015 - 2020 | Rectal swabs, nose swabs, throat swabs | Not available | Not available | 17 | 17 | Not available |

*CR-PA* carbapenem-resistant *Pseudomonas aeruginosa*, *HCW* healthcare workers, *RT-PCR* reverse transcription polymerase chain reaction.

^1^ *P. aeruginosa* isolates were only tested for the presence of VIM β-lactamase enzyme.

^2^ Numbers not available for HCW.

**References**

1. Adelantado Lacasa M, Portillo ME, Lobo Palanco J, Chamorro J, Ezpeleta Baquedano C. Molecular Epidemiology of Multidrug-Resistant *Pseudomonas aeruginosa* Acquired in a Spanish Intensive Care Unit: Using Diverse Typing Methods to Identify Clonal Types. Microorganisms. 2022;10(9). <https://doi.org/10.3390/microorganisms10091791>.

2. Catho G, Martischang R, Boroli F, Chraïti MN, Martin Y, Koyluk Tomsuk Z, et al. Outbreak of *Pseudomonas aeruginosa* producing VIM carbapenemase in an intensive care unit and its termination by implementation of waterless patient care. Crit Care. 2021;25(1):301. <https://doi.org/10.1186/s13054-021-03726-y>.

3. De Geyter D, Vanstokstraeten R, Crombé F, Tommassen J, Wybo I, Piérard D. Sink drains as reservoirs of VIM-2 metallo-β-lactamase-producing *Pseudomonas aeruginosa* in a Belgian intensive care unit: relation to patients investigated by whole-genome sequencing. J Hosp Infect. 2021;115:75-82. <https://doi.org/10.1016/j.jhin.2021.05.010>.

4. Franco R, de Oliveira Santos IC, Mora MFM, López PVA, Alvarez VET, Arce FHO, et al. Genotypic characterization and clonal relatedness of metallo-β-lactamase-producing non-fermentative gram negative bacteria in the first 5 years of their circulation in Paraguay (2011-2015). Braz J Microbiol. 2023;54(1):179-90. <https://doi.org/10.1007/s42770-022-00888-x>.

5. Freire MP, Camargo CH, Yamada AY, Nagamori FO, Reusing Junior JO, Spadão F, et al. Critical points and potential pitfalls of outbreak of IMP-1-producing carbapenem-resistant *Pseudomonas aeruginosa* among kidney transplant recipients: a case-control study. J Hosp Infect. 2021;115:83-92. <https://doi.org/10.1016/j.jhin.2021.05.006>.

6. Hu Y, Qing Y, Chen J, Liu C, Lu J, Wang Q, et al. Prevalence, Risk Factors, and Molecular Epidemiology of Intestinal Carbapenem-Resistant *Pseudomonas aeruginosa*. Microbiol Spectr. 2021;9(3):e0134421. <https://doi.org/10.1128/spectrum.01344-21>.

7. Karampatakis T, Tsergouli K, Iosifidis E, Antachopoulos C, Mouloudi E, Karyoti A, et al. Forecasting models of infections due to carbapenem-resistant Gram-negative bacteria in an intensive care unit in an endemic area. J Glob Antimicrob Resist. 2020;20:214-8. <https://doi.org/10.1016/j.jgar.2019.06.019>.

8. Maclean K, Njamo F, Serepa-Dlamini MH, Kondiah K, Green E. Antimicrobial Susceptibility Profiles among *Pseudomonas aeruginosa* Isolated from Professional SCUBA Divers with Otitis Externa, Swimming Pools and the Ocean at a Diving Operation in South Africa. Pathogens. 2022;11(1). <https://doi.org/10.3390/pathogens11010091>.

9. Mahmoud MF, Fathy FM, Gohar MK, Awad WM, Soliman MH. Genotyping of *pseudomonas aeruginosa* strains isolated from surgical site infected patients by RAPD-PCR. Syst Rev Pharm. 2020;11(12):1998-2005. <https://doi.org/10.31838/srp.2020.12.304>.

10. Martak D, Gbaguidi-Haore H, Meunier A, Valot B, Conzelmann N, Eib M, et al. High prevalence of *Pseudomonas aeruginosa* carriage in residents of French and German long-term care facilities. Clin Microbiol Infect. 2022;28(10):1353-8. <https://doi.org/10.1016/j.cmi.2022.05.004>.

11. Ohadian Moghadam S, Afshar D, Nowroozi MR, Behnamfar A, Farzin A. Molecular Epidemiology of Carbapenemase-Producing *Pseudomonas aeruginosa* Isolated from an Iranian University Hospital: Evidence for Spread of High-Risk Clones. Infect Drug Resist. 2020;13:1583-92. <https://doi.org/10.2147/idr.s253756>.

12. Odoi H, Boamah VE, Boakye YD, Agyare C. Prevalence and Phenotypic and Genotypic Resistance Mechanisms of Multidrug-Resistant *Pseudomonas aeruginosa* Strains Isolated from Clinical, Environmental, and Poultry Litter Samples from the Ashanti Region of Ghana. J Environ Public Health. 2021;2021:9976064. <https://doi.org/10.1155/2021/9976064>.

13. Pham TM, Büchler AC, Voor In 't Holt AF, Severin JA, Bootsma MCJ, Gommers D, et al. Routes of transmission of VIM-positive *Pseudomonas aeruginosa* in the adult intensive care unit-analysis of 9 years of surveillance at a university hospital using a mathematical model. Antimicrob Resist Infect Control. 2022;11(1):55. <https://doi.org/10.1186/s13756-022-01095-x>.

14. Rice W, Martin J, Hodgkin M, Carter J, Barrasa A, Sweeting K, et al. A protracted outbreak of difficult-to-treat resistant *Pseudomonas aeruginosa* in a haematology unit: a matched case-control study demonstrating increased risk with use of fluoroquinolone. J Hosp Infect. 2023;132:52-61. <https://doi.org/10.1016/j.jhin.2022.11.013>.

15. Saharman YR, Pelegrin AC, Karuniawati A, Sedono R, Aditianingsih D, Goessens WHF, et al. Epidemiology and characterisation of carbapenem-non-susceptible *Pseudomonas aeruginosa* in a large intensive care unit in Jakarta, Indonesia. Int J Antimicrob Agents. 2019;54(5):655-60. <https://doi.org/10.1016/j.ijantimicag.2019.08.003>.

16. Torrens G, van der Schalk TE, Cortes-Lara S, Timbermont L, Del Barrio-Tofiño E, Xavier BB, et al. Susceptibility profiles and resistance genomics of *Pseudomonas aeruginosa* isolates from European ICUs participating in the ASPIRE-ICU trial. J Antimicrob Chemother. 2022;77(7):1862-72. <https://doi.org/10.1093/jac/dkac122>.

17. Wendel AF, Malecki M, Mattner F, Xanthopoulou K, Wille J, Seifert H, et al. Genomic-based transmission analysis of carbapenem-resistant *Pseudomonas aeruginosa* at a tertiary care centre in Cologne (Germany) from 2015 to 2020. JAC Antimicrob Resist. 2022;4(3):dlac057. <https://doi.org/10.1093/jacamr/dlac057>.
