# Supplementary material for: In search of the best method to detect carriage of carbapenem-resistant Pseudomonas aeruginosa in humans: a systematic review
Source: Ann Clin Microbiol Antimicrob. 2024 Jun 10;23:50. doi: 10.1186/s12941-024-00707-1 (PMC11163693; doi:10.1186/s12941-024-00707-1)
Supplement: Supplementary file 6 — Supplementary Material 6. Table S5. Culture methods reported in outbreak-surveillance studies with clinical samples (n = 116). [file 12941_2024_707_MOESM6_ESM.docx]

**SUPPLEMENTARY TABLE S5: CULTURE METHODS REPORTED IN OUTBREAK-SURVEILLANCE STUDIES WITH CLINICAL SAMPLES (N=116).**

| **Culture method** | **N (%)** |
| --- | --- |
| **Study reports on type of swab** |  |
| Yes | 36 (31.0) |
| Not mentioned | 67 (57.8) |
| Not applicable | 13 (11.2) |
| *Types of swab*^a^ |  |
| Cotton swab | 28 (77.8) |
| In Cary-Blair transport medium^b^ | 1 |
| In Amies transport medium^b^ | 1 |
| Rayon swab | 3 (8.3) |
| With a semisolid transport medium | 1 |
| Nylon swab | 1 (2.8) |
| E-swab | 1 (2.8) |
| Sterile swab, not mentioned which type | 4 (11.1) |
| In Amies transport medium with charcoal^b^ | 1 |
| **Use of enrichment broth** |  |
| Yes | 9 (7.8) |
| No | 104 (89.7) |
| Not mentioned | 3 (2.6) |
| *Types of enrichment broth* |  |
| Brain heart infusion broth | 2 (22.2) |
| Tryptic soy broth (TSB) | 2 (22.2) |
| Thioglycolate broth | 2 (22.2) |
| Nutrient broth | 1 (11.1) |
| 5 mL of TSB with 2 mg/L cefotaxime and 50 mg/L vancomycin | 1 (11.1) |
| Fastidious organisms broth (in-house recipe) | 1 (11.1) |
| **Use of selective medium** |  |
| Yes | 30 (25.9) |
| No | 85 (73.3) |
| Not mentioned | 1 (0.9) |
| *Types of selective media*^a^ |  |
| (*Pseudomonas*) Cetrimide agar | 24 (80.0) |
| CHROMO agar | 1 (3.3) |
| ChromID OXA-48 | 1 (3.3) |
| CHROMagar KPC | 1 (3.3) |
| CHROMID *P. aeruginosa* | 1 (3.3) |
| MacConkey agar supplemented with 1µg/ml meropenem | 1 (3.3) |
| *Pseudomonas* agar | 1 (3.3) |
| Type not mentioned | 2 (6.7) |
| **Use of additional culture methods for carbapenemase detection** |  |
| Yes | 50 (43.1) |
| No | 65 (56.0) |
| Not mentioned | 1 (0.9) |
| *Types of additional culture methods*^a, c^ |  |
| Modified Hodge test (MHT) | 8 (16.0) |
| Combined-disk test/method | 7 (14.0) |
| Modified Carbapenem Inactivation Method (mCIM) | 7 (14.0) |
| Imipenem-EDTA combined (disk) test/method (CDST) | 7 (14.0) |
| Double Disk Synergy Test (DDST) | 7 (14.0) |
| (Rapidec) Carba-NP test | 4 (8.0) |
| MBL E-test | 4 (8.0) |
| Carbapenem Inactivation Method (CIM) | 2 (4.0) |
| Combined disk diffusion test/method | 2 (4.0) |
| Double-disk potentiation test (DDTP) | 2 (4.0) |
| Imipenem-EDTA double-disk synergy test | 2 (4.0) |
| Combined disk test (imipenem-EDTA) | 1 (2.0) |
| Imipenem-EDTA disk method | 1 (2.0) |
| Combination disk diffusion test for detection of MBLs | 1 (2.0) |
| Combination disk synergy test | 1 (2.0) |
| Combined disk synergy test (by using EDTA) | 1 (2.0) |
| Combined disk test with imipenem | 1 (2.0) |
| Combined E-test | 1 (2.0) |
| Disk diffusion imipenem-EDTA | 1 (2.0) |
| Disk potentiation test using imipenem and meropenem discs impregnated with EDTA | 1 (2.0) |
| Double disk approach (imipenem-EDTA) | 1 (2.0) |
| EDTA-modified Carbapanem Inactivation Method (eCIM) | 1 (2.0) |
| E-strips combination test | 1 (2.0) |
| Imipenem and ceftazidime resistance cloxacillin inhibition test | 1 (2.0) |
| Imipenem and meropenem disks infused by EDTA | 1 (2.0) |
| Imipenem-cloxacillin combined disk method | 1 (2.0) |
| Imipenem/doripenem combination disk test with EDTA | 1 (2.0) |
| Imipenem-EDTA combined disk synergy testing | 1 (2.0) |
| Imipenem-EDTA detection test | 1 (2.0) |
| Imipenem-EDTA double disk synergistic test | 1 (2.0) |
| Imipenem/EDTA E-test strips | 1 (2.0) |
| Imipenem-EDTA inhibition method | 1 (2.0) |
| Imipenem-EDTA test | 1 (2.0) |
| Phenotypic test with EDTA for MBL production | 1 (2.0) |
| Not mentioned | 1 (2.0) |
| **Use of NAAT for detection of carbapenemase genes (e.g., *bla_VIM_, bla_IMP_, bla_NDM_*)** |  |
| Yes | 42 (36.2) |
| No | 73 (62.9) |
| Not mentioned^d^ | 1 (0.9) |

^a^ Multiple answers per study possible.

^b^ Reported here only if specifically mentioned in the article or by personal communication with (one of) the authors.

^c^ Exact description of methods was used from the studies.

^d^ NAAT was used, but the article did not specifically mention whether this was used for the detection of carbapenem-resistance genes.
